# Stereotactic body radiation therapy in elderly patients with spinal metastases

**DOI:** 10.1007/s11060-026-05838-3

**Published:** 2026-10-16

**Authors:** Brock J. Gjesdal, Samuel Adida, Suchet Taori, Andrew H. Zureick, Serah Choi, John C. Flickinger, Pascal O. Zinn, James C. Bayley, Peter C. Gerszten, Roberta K. Sefcik

**Affiliations:** 1 Department of Neurological Surgery, University of Pittsburgh Medical Center, 200 Lothrop St Suite B-400, Pittsburgh, PA 15213 United States of America; 2 Department of Radiation Oncology, University of Pittsburgh Medical Center, 5115 Centre Ave, Pittsburgh, PA 15232 United States of America; 3 Department of Neurosurgery, Medical University of South Carolina, 96 Jonathan Lucas Street, 2060 Sam Rittenberg Boulevard, Charleston, SC 29425 United States of America

**Keywords:** Stereotactic body radiation therapy, Stereotactic radiosurgery, SBRT, SRS, Spine oncology, Elderly

## Abstract

**Purpose:**

Elderly patients with spinal metastases are often considered poor candidates for aggressive local therapy, due in part to medical comorbidity and limited evidence to guide treatment selection. This study evaluated outcomes following spine stereotactic body radiation therapy (SBRT) in this patient population and examined predictors of local failure (LF) and vertebral compression fracture (VCF).

**Methods:**

A prospectively maintained database of consecutive patients aged ≥ 70 years with available imaging who underwent spine SBRT at a quaternary referral center from 2002 to 2024 was analyzed. The median single-fraction dose was 16 Gy (range: 12–18 Gy), and multi-fraction regimens ranged from 18 to 35 Gy in 2–5 fractions.

**Results:**

Of 208 metastases in 161 patients (median age: 75 years, range: 70–94 years), 72% had prior conventional radiotherapy and 76% received a single fraction. The 6-, 12-, 24-, and 36-month cumulative incidence rates were 6%, 9%, 9%, and 10% for LF and 4%, 7%, 8%, and 9% for VCF (median follow-up: 9 months, range: 1-132 months). Gross tumor volume > 37 cc (subdistribution hazard ratio [sHR]: 6.27, 95% confidence interval [CI]: 1.48–26.63, *P* = 0.013) and Spinal Instability Neoplastic Score (SINS, per-point sHR: 1.23, 95% CI: 1.07–1.42, *P* = 0.005) were independently associated with LF; only SINS was independently associated with VCF (per-point sHR: 1.19, 95% CI: 1.06–1.34, *P* = 0.003), and 11 of 17 VCFs required cement augmentation or surgery. Age was associated with overall survival (hazard ratio: 1.05 per year, 95% CI: 1.01–1.09, *P* = 0.018); no statistically significant association was detected with LF or VCF when age was analyzed continuously, at thresholds of 75 or 80 years, or by exploratory maximally selected rank statistical analysis. Complete or partial pain response followed 61 of 148 evaluable treatments (41%) among 186 symptomatic metastases, and 18% of patients had grade 1–2 radiation toxicity.

**Conclusion:**

Spine SBRT, predominantly single-fraction reirradiation, was associated with low rates of LF and VCF; chronologic age alone should not preclude its use in appropriately selected elderly patients.

## Introduction

The incidence of spinal metastases is increasing, particularly among the elderly [1]. Spinal metastases may present with intractable pain, mechanical instability, and neurologic deficit secondary to epidural spinal cord or nerve root compression [2]. These are the principal indications for intervention, with treatment selection further guided by tumor histology and radiosensitivity, systemic disease burden, performance status, and life expectancy [3]. Although decompressive surgery can preserve or improve neurologic function, elderly patients are at increased perioperative risk [4]. Medical comorbidities and poor performance status may further limit surgical and systemic treatment options [5, 6]. With improving survival among patients with metastatic cancer, durable local control (LC) has become increasingly important in this patient population.

For elderly patients who are not operative candidates, treatment has historically consisted of palliative conventional external beam radiotherapy (cEBRT) [7]. Stereotactic body radiation therapy (SBRT) enables dose escalation while maintaining steep dose gradients that respect spinal cord constraints. Spine SBRT is associated with high rates of LC [8, 9] and multi-fraction SBRT with a higher complete pain response rate than cEBRT in a randomized trial [10], consistent with a meta-analysis of randomized trials in painful bone metastases [11]. Despite these advantages, elderly patients are underrepresented in randomized controlled trials [7, 10, 12]. and may be preferentially treated with cEBRT [13]. This treatment pattern may reflect concerns regarding reduced bone quality and an increased risk of vertebral compression fracture (VCF). Evidence regarding the safety and efficacy of spine SBRT specifically in elderly patients remains limited [14]. The present study aims to characterize outcomes in this population, with chronologic age as the primary exposure, and to identify predictors of local failure and VCF.

## Materials and methods

### Study design

A prospectively maintained database of consecutive patients with spinal metastases treated with SBRT at the University of Pittsburgh Medical Center from 2002 to 2024 was retrospectively analyzed. Patients aged ≥ 70 years at the time of treatment, the threshold used to define elderly patients in a prior study of spine SBRT [14], were eligible. Of 600 patients with 936 metastases in the database, 182 patients with 248 metastases were aged ≥ 70 years; after exclusion of 21 patients (40 metastases) without available baseline radiographic imaging, 161 patients with 208 metastases were included.

### Stereotactic body radiation therapy technique

Treatment planning and delivery evolved over the study period and have been previously described [15–18]. Selection of SBRT alone or in combination with surgical decompression and stabilization was individualized based on neurologic status, epidural disease burden, spinal instability, tumor histology, systemic disease burden, and prior treatment history, including previous cEBRT. Treatment was delivered using the CyberKnife (Accuray, Sunnyvale, CA), Synergy (Elekta, Stockholm, Sweden), and TrueBeam (Varian Medical Systems, Palo Alto, CA) platforms. Immobilization was achieved with a custom thermoplastic mask or the BodyFIX system (Elekta, Stockholm, Sweden). Image guidance was performed via 6D tracking, daily cone-beam computed tomography (CT), or ExacTrac (Brainlab, Munich, Germany). Magnetic resonance imaging (MRI) was registered to the simulation CT for target delineation, and CT myelography was utilized when metal artifact limited visualization of the neural elements. Later treatments used a clinical target volume that included the involved vertebral segment, whereas earlier treatments were often planned to the gross tumor volume (GTV). A 0–2 mm planning target volume margin was applied, excluding the spinal cord or cauda equina (contoured as the spinal canal or thecal sac plus 1 mm). Dose constraints followed institutional and published consensus guidelines at the time of treatment, and dose was prescribed most commonly to the 80% isodose line.

### Clinical definitions and treatment outcomes

Recorded patient characteristics included Karnofsky Performance Status (KPS), systemic disease burden, prior or adjuvant chemotherapy or immunotherapy, and local treatment history. Radioresistant histologies included melanoma, sarcoma, thyroid carcinoma, renal cell carcinoma, and gastrointestinal carcinomas. The Bilsky grade and Spinal Instability Neoplastic Score (SINS) were radiographically assessed for every metastasis, on preoperative imaging for metastases with prior surgery [19, 20]. For multilevel tumors treated with a single isocenter spanning ≥ 2 contiguous vertebrae, the highest Bilsky grade and highest SINS across involved vertebrae were used for analysis. Radiosurgical variables included margin dose, GTV maximum dose (D_max_), and biologically effective dose (BED_10_, α/β ratio of 10 Gy).

Patients were followed up at 1 month after SBRT, then every 3 months for 1 year, and annually thereafter, and follow-up was measured to death or, in living patients, to the last clinical evaluation by a neurosurgeon, radiation oncologist, or oncologist. Patients were generally recommended to have imaging follow-up, preferably with spine MRI, 3 months after SBRT, with further imaging protocols varying across the study period according to physician preference. Local failure (LF) was defined as radiographic tumor progression at the treated level on MRI (an increase in tumor volume or linear dimension, new or progressive epidural disease, or neurologic decline with corresponding epidural progression on MRI), as assessed by a neurosurgeon or neuroradiologist comparing pre- and post-SBRT imaging and confirmed on subsequent imaging. Pain outcomes were retrospectively evaluated at the last available clinical follow-up in patients with adequate clinical documentation and categorized as complete response (resolution of pain), partial response (improvement without resolution), stable pain, or worsened pain relative to baseline, consistent with previously published studies [21–24]. Radiation toxicity was graded using the Common Terminology Criteria for Adverse Events (CTCAE, version 5.0) [25]. VCF was defined as *de novo* fracture or progression of a baseline fracture at a treated level following SBRT, regardless of attribution; for multilevel treatments, a fracture at any treated level was counted once. All outcomes were measured from the date of SBRT to the respective event or last follow-up. Metastases without a documented LF or VCF, including those in patients who died before post-treatment imaging, were analyzed as free of that event until death, a competing event, or last follow-up.

### Statistical analysis

Statistical analyses were performed in R (version 4.5.0, R Foundation for Statistical Computing, Vienna, Austria) with the survival, cmprsk, crrSC, rms, maxstat, coxphf, mice, and sandwich packages. The cumulative incidences of LF and VCF were estimated with death treated as a competing risk. Fine-Gray models, with cluster-robust variance to account for multiple lesions per patient, were used to analyze LF and VCF per lesion, with results reported as subdistribution hazard ratios (sHRs). Overall survival (OS) was analyzed per patient from the date of first SBRT, using the characteristics of the first treated lesion, with Kaplan-Meier analysis and Cox proportional hazards models reported as hazard ratios (HRs). Stratified curves were compared using univariable Fine-Gray and Cox models, and cumulative incidence by treatment setting, platform, and fractionation was compared with Gray’s test; treatment year was analyzed continuously rather than split into eras. Radiation toxicity by fractionation was compared with logistic regression adjusted for treatment year, with variance clustered by patient.

Continuous variables were dichotomized at the median unless specified otherwise. KPS, recorded in 10-point increments, was dichotomized at > 70 (≥ 80, the median). SINS was analyzed continuously and dichotomized at > 12 (unstable). Age was analyzed primarily as a continuous variable and secondarily at clinically relevant thresholds of > 75 and > 80 years; to identify a clinically interpretable threshold, an exploratory analysis with maximally selected rank statistics searched for the age cutoff most strongly associated with each outcome between the 10th and 90th age percentiles, with *P* values corrected for this search by the exact Gaussian approximation. Multivariable models included variables significant on univariable analysis, with covariate selection further restricted to clinically relevant factors to minimize overfitting, limiting the LF and VCF models to 3 and 2 covariates, respectively, given the number of events. Analyses were complete-case. The proportional hazards assumption was assessed with Schoenfeld residuals, and a nonproportional effect was estimated separately for the first 12 months after SBRT and thereafter with a time-split Cox model.

Sensitivity analyses included cause-specific Cox models with robust variance clustered by patient, a Firth-penalized cause-specific Cox model for LF, restriction to the first treated metastasis per patient, exclusion of VCFs that occurred with or after LF, GTV and BED_10_ analyzed continuously, adjustment for prior cEBRT and treatment year, and multiple imputation of missing KPS (predictive mean matching, 20 imputations, pooled with Rubin’s rules). The associations between age and LF, OS, and VCF were further evaluated in exploratory analyses with unadjusted restricted cubic splines with knots at the 5th, 35th, 65th, and 95th age percentiles, and overall associations and nonlinearity were assessed with Wald tests, with cluster-robust variance for LF and VCF. The 95% confidence intervals (CIs) for spline estimates of LF and VCF were obtained from 300 bootstrap replicates resampling patients. Statistical significance was defined as a two-sided *P* < 0.05.

## Results

### Baseline patient and treatment characteristics

The median age at first SBRT was 75 years (range: 70–94 years), and 30 patients were aged > 80 years (19%), including 6 patients aged > 85 years (4%). Thirty-seven patients (23%) had 2 to 5 treated metastases (84 metastases). Previous local treatment included open surgical resection (31 metastases, 15%) and prior cEBRT (150 metastases, 72%). According to the SINS classification, metastases were categorized as stable (0–6, 83 metastases, 40%), potentially unstable (7–12, 101 metastases, 49%), or unstable (13–18, 24 metastases, 12%). Based on the SINS vertebral body collapse component, 73 metastases (35%) had a baseline pathologic fracture (≥ 50% collapse in 30 and < 50% collapse in 43), 62 (30%) had no collapse but > 50% vertebral body involvement, and 73 (35%) had neither; 18 metastases (9%) had undergone prior cement augmentation. Most treatments were delivered in a single fraction (158 metastases, 76%), with a median margin dose of 16 Gy (range: 12–18 Gy). Multi-fraction regimens delivered a median total margin dose of 24 Gy (range: 18–35 Gy) in 2–5 fractions (Table 1).

**Table 1:** Baseline patient and treatment characteristics for 161 elderly patients with 208 spinal metastases

Characteristic	Value
Age at first SBRT, years (per patient)	76 ± 5 [75 (70–94)]
70–74	72 (45%)
75–79	54 (34%)
80–85	29 (18%)
> 85	6 (4%)
Male sex (per patient)	99 (61%)
KPS	74 ± 11 [80 (40–100)]
Primary histology (per patient)	
Lung	41 (25%)
Prostate	25 (16%)
Renal cell	23 (14%)
Gastrointestinal	18 (11%)
Breast	16 (10%)
Thyroid	8 (5%)
Melanoma	6 (4%)
Other	24 (15%)
Radioresistant histology (per patient)	60 (37%)
Systemic disease burden (186 metastases)	
Solitary or oligometastatic (1–5 distant metastases)	151 (81%)
Polymetastatic (> 5 distant metastases)	35 (19%)
Prior or adjuvant chemotherapy	125 (60%)
Prior or adjuvant immunotherapy	26 (13%)
Prior open surgical resection	31 (15%)
Prior cEBRT	150 (72%)
Pain at presentation	186 (89%)
Paraspinal soft-tissue involvement	79 (38%)
Spinal region	
Cervical	24 (12%)
Thoracic	98 (47%)
Lumbar	54 (26%)
Sacral	24 (12%)
Junctional (spanning two regions)	8 (4%)
Multilevel lesion (≥ 2 vertebrae)	59 (28%)
Bilsky grade	
0	104 (50%)
1a	21 (10%)
1b	28 (13%)
1c	19 (9%)
2	27 (13%)
3	9 (4%)
Epidural disease (Bilsky grade > 0)	104 (50%)
Bone lesion	
Blastic	41 (20%)
Mixed	46 (22%)
Lytic	121 (58%)
SINS	
Stable (0–6)	83 (40%)
Potentially unstable (7–12)	101 (49%)
Unstable (13–18)	24 (12%)
Number of treated vertebral levels	1 ± 1 [1 (1–6)]
GTV (cc)	50 ± 46 [37 (2-263)]
Year of treatment	
2002–2010	133 (64%)
2011–2017	29 (14%)
2018–2024	46 (22%)
Treatment platform	
CyberKnife	105 (50%)
Synergy	38 (18%)
TrueBeam	65 (31%)
Single-fraction regimen	158 (76%)
Margin dose (Gy)	16 ± 1 [16 (12–18)]
GTV D_max_ (Gy)	19 ± 2 [20 (13–26)]
Multi-fraction regimen	50 (24%)
Fractions	3 ± 1 [3 (2–5)]
Margin dose (Gy)	24 ± 3 [24 (18–35)]
GTV D_max_ (Gy)	28 ± 3 [29 (20–35)]
BED_10_ (Gy)	42 ± 7 [42 (26–60)]

**BED**_**10**_, biologically effective dose assuming an α/β ratio of 10 Gy; **cc**, cubic centimeters; **cEBRT**, conventional external beam radiation therapy; **D**_**max**_, maximum dose; **GTV**, gross tumor volume; **Gy**, gray; **KPS**, Karnofsky Performance Status; **SBRT**, stereotactic body radiation therapy; **SINS**, Spinal Instability Neoplastic Score. Values are presented per lesion and as number (%) or average ± standard deviation [median (range)] unless specified otherwise. Radioresistant histologies included melanoma, sarcoma, thyroid carcinoma, renal cell carcinoma, and gastrointestinal carcinomas. Systemic disease burden at the time of treatment was available for 186 of 208 treatments. Data were available for margin dose in 123 of 158 single-fraction and 49 of 50 multi-fraction treatments, GTV D_max_ in 156 of 158 and 32 of 50, GTV in 194 of 208, BED_10_ in 172 of 208, and KPS in 179 of 208 treatments (136 of 161 patients); multi-fraction doses are totals, and KPS is recorded in 10-point increments. Other primary malignancies were multiple myeloma (7), bladder or urothelial carcinoma (6), sarcoma (5), head and neck carcinoma (4), endometrial carcinoma (1), and pheochromocytoma (1); the 5 sarcomas are included among radioresistant histologies. The most common regimens were 16 Gy (59 treatments), 18 Gy (18), 14 Gy (14), and 15 Gy (10) in 1 fraction and 24 Gy in 3 (12) or 2 (10) fractions

### Clinical and radiographic outcomes

**Fig. 1:**
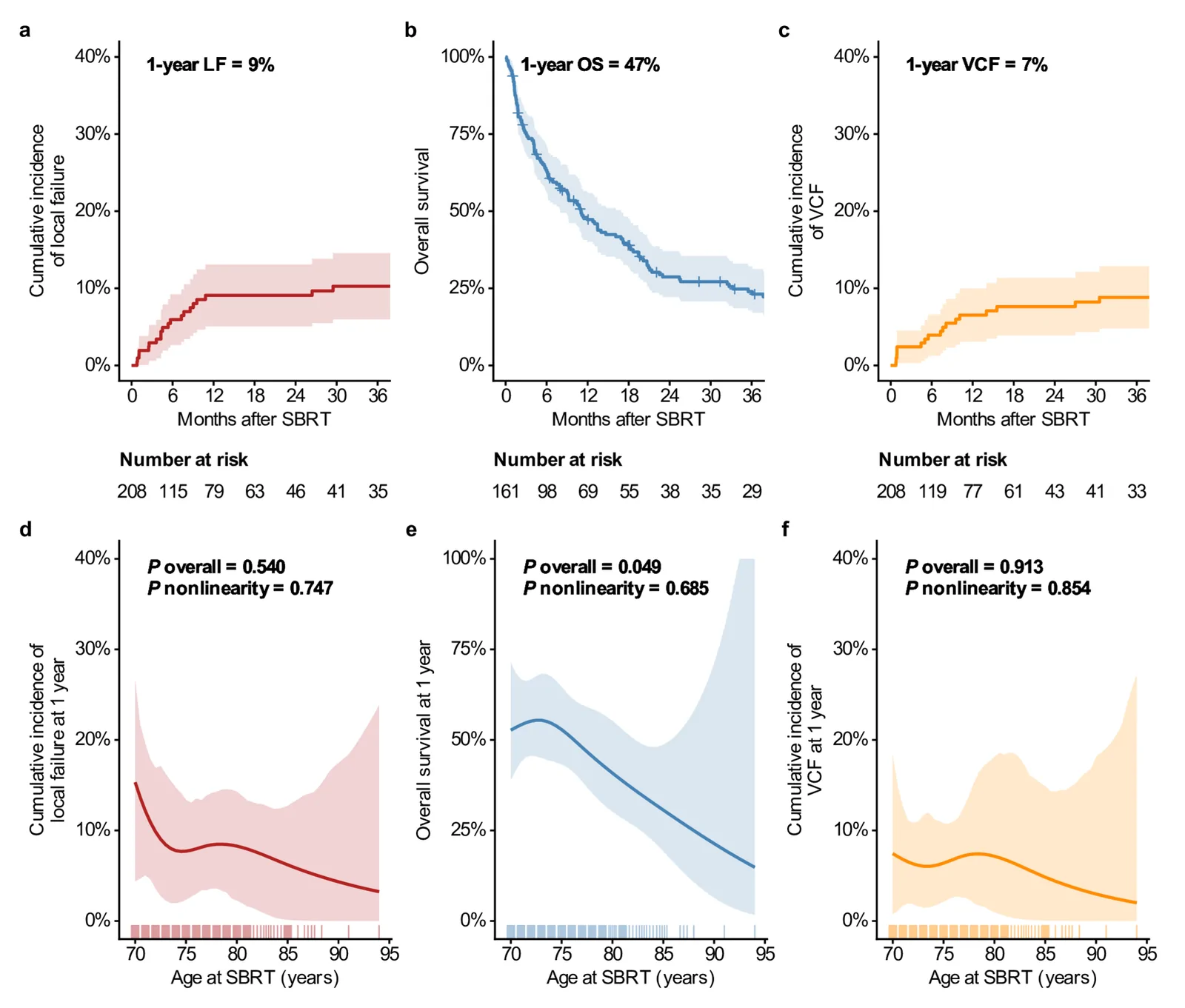
Cumulative incidence of local failure (**a**), Kaplan-Meier estimate of overall survival (**b**), and cumulative incidence of vertebral compression fractures (**c**), with 95% confidence bands and numbers at risk every 6 months; + marks in (**b**) indicate censored patients. Cumulative incidence was estimated with death as a competing risk. One-year outcomes were modeled continuously as a function of age with unadjusted restricted cubic splines (knots at 70, 73, 77, and 85 years) for local failure (**d**), overall survival (**e**), and vertebral compression fractures (**f**), using Fine-Gray (**d**, **f**) and Cox (**e**) models; shaded bands are 95% confidence intervals from 300 patient-level bootstrap resamples (**d**, **f**) or the Cox model (**e**), and tick marks show the age distribution of treated metastases (**d**, **f**) and patients (**e**). *P* values in (**d**), (**e**), and (**f**) are from Wald tests of the overall association between age and each outcome and of nonlinearity, with cluster-robust variance in (**d**) and (**f**). LF, local failure; OS, overall survival; SBRT, stereotactic body radiation therapy; VCF, vertebral compression fracture

After a median follow-up of 9 months (range: 1-132 months; median 18 months among the 23 patients alive at last follow-up), the 1- and 3-year cumulative incidence rates of LF were 9% and 10%, respectively (Table 2; Fig. 1). Median OS was 11 months, with 1- and 3-year OS rates of 47% and 24%, respectively. Among 186 metastases presenting with pain, pain response was evaluable following 148 treatments, with complete or partial responses observed following 61 treatments (41%, or 33% of all symptomatic metastases). Grade 1–2 radiation toxicity occurred in 29 patients (18%) after 34 treatments, with 39 events, most commonly pain flare (23 events, of which 19 were grade 2 and managed with corticosteroids). No radiation myelopathy or radiculopathy and, apart from VCF, no grade 3 or higher toxicity occurred. The crude rate of VCF was 8% (17 VCFs, 10 *de novo* and 7 progressive). Four VCFs occurred in metastases that also had LF, 2 at the time of progression and 2 after it. The 1- and 3-year cumulative incidence rates of VCF were 7% and 9%, respectively. Grade 3 VCFs were managed with percutaneous cement augmentation (7 VCFs) or open surgical decompression with or without instrumented fusion (4 VCFs). Eight metastases in 7 patients were treated at age > 85 years, including 1 patient first treated at age 85 and again at 86; none developed LF or VCF, and all 7 patients died a median of 11 months (range: 1–20 months) after their first treatment at age > 85 years.

**Table 2:** Clinical and radiographic outcomes for elderly patients treated with spine stereotactic body radiation therapy

Characteristic	Value
Follow-up (months)	19 ± 26 [9 (1-132)]
Local tumor control	
Crude rate of local failure	21 (10%)
Time to local failure (months)	9 ± 11 [5 (1–43)]
Cumulative incidence of local failure	
6-month	6% (95% CI: 3–9%)
12-month	9% (95% CI: 5–13%)
24-month	9% (95% CI: 5–13%)
36-month	10% (95% CI: 6–15%)
Metastases at risk at 6 / 12 / 24 / 36 months	115 / 79 / 46 / 35
Overall survival	
Time to death or last follow-up (months, per patient)	19 ± 26 [9 (1-132)]
Median overall survival (months)	11
Kaplan-Meier estimates of overall survival	
6-month	63% (95% CI: 56–71%)
12-month	47% (95% CI: 40–56%)
24-month	29% (95% CI: 22–37%)
36-month	24% (95% CI: 18–32%)
Patients at risk at 6 / 12 / 24 / 36 months	98 / 69 / 38 / 29
Pain response (186 symptomatic metastases; 148 evaluable)	
Complete	25 (17%)
Partial	36 (24%)
Stable	55 (37%)
Worsened	32 (22%)
Not evaluable	38 of 186 (20%)
Died before first follow-up	26
Not documented	12
Radiation toxicity	
Time to radiation toxicity (months)	1 ± 2 [1 (1–7)]
Patients with radiation toxicity	29 (18%)
Treatments with radiation toxicity	34 (16%)
Toxicity events	39
Events by type	
Pain flare	23 (19 grade 2)
Fatigue	6
Nausea	4
Esophagitis	2
Pneumonitis	1
Other	3
Maximum CTCAE grade per treatment	
Grade 1	15 (7%)
Grade 2	19 (9%)
VCF	
Crude rate of VCF	17 (8%)
Time to VCF (months)	9 ± 9 [7 (1–31)]
Cumulative incidence of VCF	
6-month	4% (95% CI: 1–7%)
12-month	7% (95% CI: 3–10%)
24-month	8% (95% CI: 4–11%)
36-month	9% (95% CI: 5–13%)
Metastases at risk at 6 / 12 / 24 / 36 months	119 / 77 / 43 / 33
CTCAE grade (17 VCFs)	
Grade 2	6 (35%)
Grade 3	11 (65%)
Outcomes by treatment setting (de novo / reirradiation / postoperative)	
Metastases	53 / 124 / 31
12-month cumulative incidence of local failure	8% / 9% / 10% (*P* = 0.434)
12-month cumulative incidence of VCF	4% / 7% / 7% (*P* = 0.874)
Treatments with radiation toxicity	7 (13%) / 21 (17%) / 6 (19%)
Complete or partial pain response	16 (41%) / 39 (45%) / 6 (26%)

**cEBRT**, conventional external beam radiation therapy; **CI**, confidence interval; **CTCAE**, Common Terminology Criteria for Adverse Events; **VCF**, vertebral compression fracture. Values are presented per lesion and as number (%) or average ± standard deviation [median (range)] unless specified otherwise. Cumulative incidence analysis for local failure and vertebral compression fracture was performed with death as a competing risk. Pain response was available for 148 of 186 metastases (80%) presenting with pain. Radiation toxicity was graded according to the Common Terminology Criteria for Adverse Events (version 5.0, National Cancer Institute). Numbers at risk are metastases (local failure, VCF) or patients (overall survival) remaining under observation without the event at 6, 12, 24, and 36 months. Radiation toxicity is reported per patient, per treatment, or per event as labeled; the grade distribution uses the maximum grade per treatment, and other events were dysphagia, radiation dermatitis, and skin sensitivity (1 each). Treatment setting: de novo, no prior open surgical resection or cEBRT at the treated level; reirradiation, prior cEBRT at the treated level without prior open surgical resection; postoperative, prior open surgical resection at the treated level (26 of 31 also previously irradiated). Toxicity by setting is per treatment, and pain response by setting is among evaluable symptomatic metastases (39, 86, and 23). Cumulative incidence by treatment setting was compared with Gray’s test

LF and VCF did not differ significantly by treatment setting (de novo, reirradiation, or postoperative; *P* = 0.434 and 0.874, respectively; Table 2). LF was not significantly associated with treatment year (*P* = 0.689) and did not differ significantly by platform or fractionation (*P* = 0.870 and 0.604, respectively). VCF risk increased with later treatment year (sHR: 1.56 per 5 years, 95% CI: 1.09–2.24, *P* = 0.016) and was higher after linear accelerator treatment (*P* = 0.043) but did not differ significantly by fractionation (*P* = 0.118); all treatments after 2016 were delivered with gantry-based linear accelerators, and most were multi-fraction. Prior or adjuvant immunotherapy became more common over time (7 of 133 treatments in 2002–2010 and 16 of 46 in 2018–2024). Radiation toxicity was recorded after 20 of 50 multi-fraction and 14 of 158 single-fraction treatments, a difference that was not statistically significant after adjustment for treatment year (*P* = 0.212).

Restricted cubic splines estimated 1-year outcomes continuously as a function of age. On spline analysis, estimated 1-year OS decreased with increasing age (*P* for overall association = 0.049), with no evidence of nonlinearity (*P* for nonlinearity = 0.685), consistent with the association of continuous age with OS (Table 3). No statistically significant association was detected between age and LF or VCF on spline analysis (*P* for overall association = 0.540 and 0.913, respectively; Fig. 1).

**Table 3:** Univariable and multivariable analysis of local failure, overall survival, and vertebral compression fracture

	Univariable			Multivariable		
	HR or sHR	95% CI	*P*	HR or sHR	95% CI	*P*
Local failure						
Age						
Continuous, per year	0.95	0.86–1.05	0.296	-	-	-
> 71 years*	0.56	0.23–1.38	0.864	-	-	-
> 75 years	0.77	0.34–1.73	0.527	-	-	-
> 80 years	0.72	0.23–2.23	0.568	-	-	-
Male sex	2.27	0.89–5.79	0.088	-	-	-
KPS > 70	0.61	0.25–1.47	0.268	-	-	-
Radioresistant histology	2.34	1.00-5.48	0.050	-	-	-
Polymetastatic disease	1.52	0.50–4.63	0.459	-	-	-
Prior or adjuvant chemotherapy	0.47	0.20–1.14	0.095	-	-	-
Prior or adjuvant immunotherapy	1.19	0.36–3.93	0.775	-	-	-
Prior cEBRT	1.21	0.47–3.08	0.694	-	-	-
Prior open surgical resection	1.86	0.76–4.56	0.177	-	-	-
Lytic bone lesion	7.38	1.79–30.37	**0.006**	-	-	-
Paraspinal soft-tissue involvement	2.90	1.21–6.93	**0.017**	-	-	-
High-grade epidural disease	6.30	2.60-15.27	**< 0.001**	2.47	0.79–7.69	0.119
SINS						
Continuous, per point	1.37	1.22–1.54	**< 0.001**	1.23	1.07–1.42	**0.005**
> 12	5.82	2.33–14.52	**< 0.001**	-	-	-
GTV > 37 cc	9.93	2.33–42.28	**0.002**	6.27	1.48–26.63	**0.013**
BED_10_ > 42 Gy	1.58	0.55–4.55	0.393	-	-	-
Overall survival						
Age						
Continuous, per year	1.05	1.01–1.09	**0.009**	1.05	1.01–1.09	**0.018**
> 75 years	1.50	1.06–2.11	**0.022**	-	-	-
> 76 years*	1.62	1.13–2.30	0.088	-	-	-
> 80 years	1.77	1.15–2.72	**0.010**	-	-	-
Male sex	1.26	0.89–1.79	0.201	-	-	-
KPS > 70	0.56	0.39–0.82	**0.002**	0.56	0.38–0.81	**0.002**
Radioresistant histology	1.01	0.71–1.43	0.973	-	-	-
Polymetastatic disease	0.90	0.55–1.45	0.655	-	-	-
Prior or adjuvant chemotherapy	0.77	0.54–1.09	0.137	-	-	-
Prior or adjuvant immunotherapy	0.80	0.47–1.35	0.400	-	-	-
Prior cEBRT	1.91	1.28–2.87	**0.002**	1.98	1.26–3.09	**0.003**
Prior open surgical resection	1.32	0.83–2.09	0.236	-	-	-
Lytic bone lesion	0.88	0.63–1.24	0.481	-	-	-
Paraspinal soft-tissue involvement	1.33	0.95–1.88	0.099	-	-	-
High-grade epidural disease	1.35	0.86–2.12	0.196	-	-	-
SINS						
Continuous, per point	1.01	0.96–1.07	0.686	-	-	-
> 12	1.00	0.58–1.71	1.000	-	-	-
GTV > 37 cc	1.31	0.93–1.84	0.122	-	-	-
BED_10_ > 42 Gy	0.86	0.57–1.29	0.469	-	-	-
Vertebral compression fracture						
Age						
Continuous, per year	0.98	0.88–1.09	0.751	-	-	-
> 75 years	0.91	0.33–2.53	0.863	-	-	-
> 80 years	1.37	0.37–5.08	0.643	-	-	-
Male sex	0.59	0.22–1.62	0.308	-	-	-
KPS > 70	0.92	0.33–2.58	0.873	-	-	-
Radioresistant histology	1.97	0.73–5.33	0.182	-	-	-
Polymetastatic disease	1.39	0.48–4.08	0.545	-	-	-
Prior or adjuvant chemotherapy	2.05	0.65–6.44	0.221	-	-	-
Prior or adjuvant immunotherapy	1.54	0.45–5.27	0.490	-	-	-
Prior cEBRT	0.68	0.25–1.90	0.466	-	-	-
Prior open surgical resection	0.76	0.18–3.29	0.715	-	-	-
Lytic bone lesion	2.42	0.78–7.53	0.128	-	-	-
Paraspinal soft-tissue involvement	1.47	0.54–4.02	0.455	-	-	-
High-grade epidural disease	2.84	1.00-8.07	**0.0498**	1.41	0.47–4.24	0.546
SINS						
Continuous, per point	1.22	1.08–1.37	**< 0.001**	1.19	1.06–1.34	**0.003**
> 12	2.59	0.81–8.26	0.109	-	-	-
GTV > 37 cc	1.02	0.36–2.85	0.973	-	-	-
BED_10_ > 42 Gy	1.80	0.59–5.50	0.305	-	-	-

**BED**_**10**_, biologically effective dose assuming an α/β ratio of 10 Gy; **cc**, cubic centimeters; **cEBRT**, conventional external beam radiation therapy; **CI**, confidence interval; **GTV**, gross tumor volume; **Gy**, gray; **HR**, hazard ratio; **KPS**, Karnofsky Performance Status; **sHR**, subdistribution hazard ratio; **SINS**, Spinal Instability Neoplastic Score. Fine-Gray subdistribution hazard models were utilized to estimate local failure and vertebral compression fracture per lesion, with results reported as subdistribution hazard ratios. Fine-Gray models used a cluster-robust variance estimator with patient as the cluster. Overall survival was estimated per patient using Kaplan-Meier analysis and evaluated using Cox proportional hazards models, with results reported as hazard ratios. Multivariable models included variables significant on univariable analysis, with covariate selection further restricted to clinically relevant factors to minimize overfitting. Model sizes were 194 lesions in 152 patients with 20 events (local failure), 136 patients with 115 deaths (overall survival), and 208 lesions with 17 events (vertebral compression fracture). Events by stratum were, for local failure, 2 of 97 metastases with GTV ≤ 37 cc and 18 of 97 with GTV > 37 cc, 10 of 172 with Bilsky grade 0–1 and 11 of 36 with grade 2 or 3, and 13 of 184 with SINS ≤ 12 and 8 of 24 with SINS > 12; for vertebral compression fracture, 11 of 172 and 6 of 36 by Bilsky grade and 13 of 184 and 4 of 24 by SINS, respectively. High-grade epidural disease was defined as Bilsky grade 2 or 3. Radioresistant histologies included melanoma, sarcoma, thyroid carcinoma, renal cell carcinoma, and gastrointestinal carcinomas. Because KPS is recorded in 10-point increments, KPS > 70 is equivalent to KPS ≥ 80, the cohort median. Bold *P* values indicate *P* < 0.05. *Maximally selected rank statistics were used in an exploratory analysis to identify cutoffs of age > 71 years for local failure, age > 76 years for overall survival, and age > 80 years for vertebral compression fracture (also a clinical threshold; corrected *P* = 0.880). *P* values for the exploratory cutoffs are corrected for the search across cutoffs (exact Gaussian approximation); for local failure and vertebral compression fracture, the search used log-rank tests that treated death as censoring

### Predictors of local failure, overall survival, and vertebral compression fracture

No statistically significant association was detected between age and LF, whether age was analyzed continuously (sHR: 0.95 per year, 95% CI: 0.86–1.05, *P* = 0.296), at thresholds of > 75 or > 80 years, or at the exploratory maximally selected cutoff of > 71 years. On univariable analysis, LF was associated with high-grade epidural disease (Bilsky grade 2 or 3), lytic bone lesions, GTV > 37 cc, SINS, and paraspinal soft-tissue involvement (Table 3). On multivariable analysis, GTV > 37 cc (sHR: 6.27, 95% CI: 1.48–26.63, *P* = 0.013) and SINS (sHR: 1.23 per point, 95% CI: 1.07–1.42, *P* = 0.005) remained associated with LF.

On univariable analysis, age was associated with OS when analyzed continuously (HR: 1.05 per year, 95% CI: 1.01–1.09, *P* = 0.009) and at thresholds of > 75 and > 80 years. In the exploratory analysis, maximally selected rank statistics identified age > 76 years as the data-derived cutoff for OS, although it was not statistically significant (corrected *P* = 0.088). On multivariable analysis, age (HR: 1.05 per year, 95% CI: 1.01–1.09, *P* = 0.018), KPS > 70 (HR: 0.56, 95% CI: 0.38–0.81, *P* = 0.002), and prior cEBRT (HR: 1.98, 95% CI: 1.26–3.09, *P* = 0.003) were independently associated with OS, including after multiple imputation of missing KPS. The effect of age was not proportional over time: age was not significantly associated with OS during the first 12 months after SBRT (*P* = 0.662) but was thereafter (HR: 1.14 per year, 95% CI: 1.07–1.22, *P* < 0.001). The proportional hazards assumption was otherwise met.

No statistically significant association was detected between age and VCF, whether age was analyzed continuously (sHR: 0.98 per year, 95% CI: 0.88–1.09, *P* = 0.751) or at thresholds of > 75 or > 80 years (the latter also the exploratory maximally selected cutoff). On univariable analysis, SINS and high-grade epidural disease were associated with VCF, and, on multivariable analysis, SINS remained associated with VCF (sHR: 1.19 per point, 95% CI: 1.06–1.34, *P* = 0.003) (Table 3). The multivariable associations for LF and VCF were consistent across all sensitivity analyses, and no statistically significant association between age and LF or VCF was detected in cause-specific models or after restriction to the first treated metastasis per patient.

**Fig. 2:**
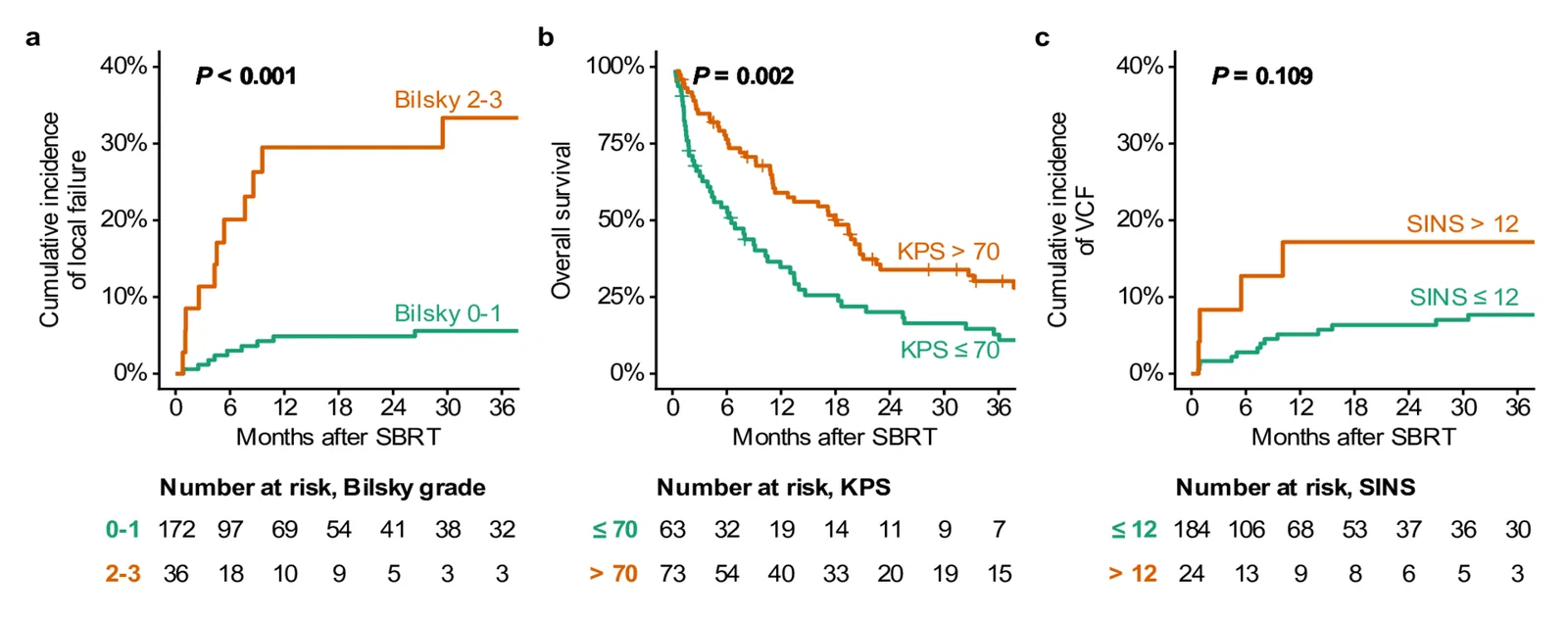
Cumulative incidence of local failure by Bilsky grade (**a**), Kaplan-Meier estimate of overall survival by Karnofsky Performance Status (KPS) (**b**), and cumulative incidence of vertebral compression fractures by Spinal Instability Neoplastic Score (SINS) (**c**). Cumulative incidence was estimated with death as a competing risk. Numbers at risk are shown every 6 months beneath each panel; + marks in (**b**) indicate censored patients, and KPS was unavailable for 25 patients. *P* values are from univariable Fine-Gray models with cluster-robust variance (**a**, **c**) and a univariable Cox model (**b**). SBRT, stereotactic body radiation therapy; VCF, vertebral compression fracture

LF was more frequent among metastases with high-grade epidural disease (*P* < 0.001), OS was superior among patients with KPS > 70 (*P* = 0.002), and VCF did not differ significantly by SINS > 12 (*P* = 0.109) (Fig. 2).

## Discussion

Elderly patients represent a growing proportion of patients with spinal metastases, yet remain underrepresented in studies reporting on outcomes following spine SBRT [1, 14]. The present cohort included 161 patients aged ≥ 70 years, with 19% of patients aged > 80 years; 72% of metastases had prior cEBRT, and 76% were treated in a single fraction. The cumulative incidence of LF was 9% at 1 year and 10% at 3 years, with death as a competing event; because most patients died within 2 years, these rates do not represent the proportion of patients alive with controlled disease. Complete or partial pain response followed 41% of evaluable treatments, and no radiation myelopathy or radiculopathy occurred, but 11 of 17 VCFs required cement augmentation or surgery. The outcomes reported in this study are comparable to those reported in larger cohorts [8, 26–29], including series limited to traditionally radioresistant histologies [22, 24, 30–34]. and those evaluating SBRT in the setting of reirradiation [35–39]. These findings support the use of spine SBRT in selected elderly patients and suggest that chronologic age alone should not preclude treatment.

Although spine cEBRT provides effective pain palliation, recurrent pain and local tumor progression remain important limitations [40, 41]. Local tumor control in patients with spinal metastases improves functional independence and quality of life [42, 43]. This has been increasingly important as advances in targeted and immunotherapies prolong survival [44]. The randomized evidence on pain response differs by regimen: SC.24 found a higher complete response rate with 24 Gy in 2 fractions than with cEBRT [10], whereas NRG/RTOG 0631 found no improvement in pain response with 16–18 Gy in 1 fraction compared with 8 Gy in 1 fraction [7]. Although not directly comparable with the 3-month trial endpoints, the 17% complete response rate at last follow-up in this predominantly single-fraction cohort is more consistent with the latter. Worsened pain often accompanied LF or VCF: 15 of the 32 metastases with worsened pain had LF or VCF at the treated level. These considerations are particularly relevant in elderly patients, where LC must be considered alongside treatment morbidity and expected survival.

LF and VCF were associated with tumor and mechanical characteristics, whereas no statistically significant association was detected between age and LF or VCF with any parameterization of age or in cause-specific models, which do not treat death as a competing event. Larger tumor volume and spinal instability were associated with LF, consistent with prior studies [21, 29, 45]. These associations may reflect greater local disease burden and challenges in achieving adequate dose coverage. The GTV estimate was imprecise, as only 2 LF events occurred among metastases with GTV ≤ 37 cc. Age, performance status, and prior cEBRT were associated with OS, which is consistent with known prognostic factors in patients with metastatic disease [46, 47]. The association of prior cEBRT with shorter survival may reflect a longer and more advanced disease course rather than harm from reirradiation. The prognostic significance of age and performance status has been demonstrated in recursive partitioning analyses of patients undergoing spine SBRT [48]. This study also found that spinal instability was associated with VCF, consistent with evidence demonstrating increased VCF risk with higher SINS [49, 50]. Because vertebral body collapse contributes to SINS and progression of a baseline fracture was counted as VCF, this association may partly reflect pre-existing structural compromise. Previous analyses from this institution found that age > 61 years was not associated with increased local tumor progression and was not significantly associated with OS [27], while a separate series found no association between age and VCF [51].

LF and VCF did not differ significantly between de novo, reirradiation, and postoperative treatment, but these results reflect a selected population treated at a quaternary referral center, mostly with single-fraction SBRT for previously irradiated metastases, and should be interpreted in that context, as many elderly patients with spinal metastases have poorer performance status, greater comorbidity, or more extensive disease than patients selected for SBRT.

In a recent series of 252 patients aged ≥ 70 years, Chan et al. reported increasing age as an independent predictor of VCF, with the greatest risk among patients aged ≥ 86 years [14]. No statistically significant association was detected in the present cohort, possibly because few patients were aged > 85 years. Chan et al. also identified higher dose as a predictor of fracture [14]. In the present cohort, BED_10_ was not significantly associated with LF or VCF, but its narrow interquartile range (38–46 Gy) means that a dose effect cannot be excluded. Antiresorptive therapy may represent an additional strategy for mitigating fracture risk, with recent evidence demonstrating lower rates of VCF among patients receiving bisphosphonates or denosumab prior to spine SBRT [52–54]. Prophylactic cement augmentation with kyphoplasty prior to SBRT may also be considered in selected patients and has been shown to be safe without compromising treatment planning or delivery [55, 56].

### Limitations

This single-institution retrospective study is subject to selection bias. The median follow-up of 9 months reflects the limited survival of this elderly population and may restrict assessment of LF and late radiation toxicity, including VCFs. Because of competing mortality, estimates beyond 2 years rest on few metastases at risk. Pain outcomes were retrospectively assessed, did not account for analgesic use, and could not be evaluated using standardized quantitative measures due to record heterogeneity. No comorbidity index, frailty measure, or geriatric assessment was available, so frailty, which was independently associated with inferior LC and OS but not VCF after spine SBRT in a prior institutional series [57], could not be accounted for. Histologies and systemic therapies were heterogeneous, and treatment year, platform, and fractionation changed together over two decades and could not be reliably separated; multi-fraction treatments were few. In patients treated with cEBRT before SBRT, the timing and dose of prior radiation were not available for every patient and could not be accounted for in the analysis. Radiographic endpoints were censored at the date of last clinical follow-up rather than the date of last evaluable imaging, which may have underestimated the incidence of LF and VCF. Failure location relative to the treated volume and treatment-related imaging changes were not recorded systematically; Spine Response Assessment in Neuro-Oncology (SPINO) criteria were not formally applied; and bone density, antiresorptive therapy, prior spinal instrumentation, neurologic status at presentation, and details of surgical evaluation were not consistently available. The low incidence of LF and VCF limited the number of covariates in multivariable models and the precision of estimates, and a modest effect of age cannot be excluded. Patients aged > 85 years comprised 4% of the cohort, limiting the ability to evaluate the increased VCF risk reported by Chan et al. in this age group [14]. Multi-institutional studies are warranted to validate these findings and further characterize outcomes following spine SBRT in the elderly.

## Conclusion

Spine stereotactic body radiation therapy, predominantly single-fraction reirradiation, was associated with low rates of local failure and vertebral compression fracture in appropriately selected elderly patients with spinal metastases; although fractures were uncommon, most required intervention. Treatment selection should be guided by tumor characteristics, spinal stability, and overall prognosis rather than chronologic age alone. 

## Data Availability

The dataset is available from the corresponding author upon reasonable request.

## References

[1] Shakil H, Malhotra AK, Badhiwala JH, Karthikeyan V, Essa A, He Y et al (2024) Contemporary trends in the incidence and timing of spinal metastases: A population-based study. Neuro-Oncology Adv 6(1):vdae051 10.1093/noajnl/vdae051 PMC11046986 38680988

[2] Van den Brande R, Billiet C, Peeters M, Van de Kelft E (2024) Spinal Metastases of the Vertebrae: Three Main Categories of Pain. Life 14(8):988 10.3390/life14080988 PMC11355794 39202730

[3] Laufer I, Rubin DG, Lis E, Cox BW, Stubblefield MD, Yamada Y et al (2013) The NOMS framework: approach to the treatment of spinal metastatic tumors. Oncologist 18(6):744–751 10.1634/theoncologist.2012-0293 PMC4063402 23709750

[4] Amelot A, Balabaud L, Choi D, Fox Z, Crockard HA, Albert T et al (2017) Surgery for metastatic spine tumors in the elderly. Advanced age is not a contraindication to surgery! Spine J 17(6):759–767 10.1016/j.spinee.2015.07.440 26239762

[5] Craigs CL, Bennett MI, Hurlow A, West RM, Ziegler LE (2018) Older age is associated with less cancer treatment: a longitudinal study of English cancer patients. Age Ageing 47(6):833–840 10.1093/ageing/afy094 29982340

[6] Liang T, Wan Y, Zou X, Peng X, Liu S (2013) Is surgery for spine metastasis reasonable in patients older than 60 years? Clin Orthop Relat Res 471(2):628–639 10.1007/s11999-012-2699-3 PMC3549148 23179121

[7] Ryu S, Deshmukh S, Timmerman RD, Movsas B, Gerszten P, Yin FF et al (2023) Stereotactic radiosurgery vs conventional radiotherapy for localized vertebral metastases of the spine: Phase 3 results of NRG oncology/RTOG 0631 randomized clinical trial. JAMA Oncol 9(6):800–807 10.1001/jamaoncol.2023.0356 PMC10119775 37079324

[8] Guckenberger M, Andratschke N, Belka C, Bellut D, Cuccia F, Dahele M et al (2024) ESTRO clinical practice guideline: Stereotactic body radiotherapy for spine metastases. Radiother Oncol 190:109966 10.1016/j.radonc.2023.109966 37925107

[9] Zeng KL, Myrehaug S, Soliman H, Husain ZA, Tseng CL, Detsky J et al (2022) Mature Local Control and Reirradiation Rates Comparing Spine Stereotactic Body Radiation Therapy With Conventional Palliative External Beam Radiation Therapy. Int J Radiat Oncol Biol Phys 114(2):293–300 10.1016/j.ijrobp.2022.05.043 35675854

[10] Sahgal A, Myrehaug SD, Siva S, Masucci GL, Maralani PJ, Brundage M et al (2021) Stereotactic body radiotherapy versus conventional external beam radiotherapy in patients with painful spinal metastases: An open-label, multicentre, randomised, controlled, phase 2/3 trial. Lancet Oncol 22(7):1023–1033 10.1016/S1470-2045(21)00196-0 34126044

[11] Bindels BJJ, Mercier C, Gal R, Verlaan JJ, Verhoeff JJC, Dirix P et al (2024) Stereotactic Body and Conventional Radiotherapy for Painful Bone Metastases: A Systematic Review and Meta-Analysis. JAMA Netw Open 7(2):e2355409 10.1001/jamanetworkopen.2023.55409 PMC10862159 38345820

[12] Sprave T, Verma V, Förster R, Schlampp I, Bruckner T, Bostel T et al (2018) Randomized phase II trial evaluating pain response in patients with spinal metastases following stereotactic body radiotherapy versus three-dimensional conformal radiotherapy. Radiother Oncol 128(2):274–282 10.1016/j.radonc.2018.04.030 29843899

[13] Ong WL, Milne RL, Foroudi F, Millar JL (2023) Stereotactic Body Radiation Therapy for Spine Metastases-Findings from an Australian Population-Based Study. Curr Oncol 30(8):7777–7788 10.3390/curroncol30080564 PMC10453727 37623045

[14] Chan AW, Zeng KL, Moore-Palhares D, Atenafu EG, Chen H, Myrehaug S et al (2025) Spine stereotactic body radiation therapy in geriatric patients: Implications of age and dose on iatrogenic vertebral compression fracture risk. Int J Radiat Oncol Biol Phys 121(5):1185–1193 10.1016/j.ijrobp.2024.11.082 39580002

[15] Gerszten PC, Burton SA, Ozhasoglu C, Welch WC (2007) Radiosurgery for spinal metastases: Clinical experience in 500 cases from a single institution. Spine 32(2):193–199 10.1097/01.brs.0000251863.76595.a2 17224814

[16] Gerszten PC, Monaco EA 3rd, Quader M, Novotny J Jr, Kim JO, Flickinger JC et al (2010) Setup accuracy of spine radiosurgery using cone beam computed tomography image guidance in patients with spinal implants. J Neurosurgery: Spine 12(4):413–420 10.3171/2009.10.SPINE09249 20367378

[17] Ling DC, Flickinger JC, Burton SA, Heron DE, Quinn AE, Bejjani GK et al (2018) Long-Term Outcomes After Stereotactic Radiosurgery for Spine Metastases: Radiation Dose-Response for Late Toxicity. Int J Radiat Oncol Biol Phys 101(3):602–609 10.1016/j.ijrobp.2018.02.035 PMC7453968 29678526

[18] Adida S, Taori S, Bhatia S, Kann MR, Burton SA, Flickinger JC et al (2025) A case series and review of stereotactic body radiation therapy for contiguous multilevel spine metastases. J Neurooncol 171(2):299–309 10.1007/s11060-024-04863-4 39527381

[19] Bilsky MH, Laufer I, Fourney DR, Groff M, Schmidt MH, Varga PP et al (2010) Reliability analysis of the epidural spinal cord compression scale. J Neurosurgery: Spine 13(3):324–328 10.3171/2010.3.SPINE09459 20809724

[20] Fisher CG, DiPaola CP, Ryken TC, Bilsky MH, Shaffrey CI, Berven SH et al (2010) A novel classification system for spinal instability in neoplastic disease: An evidence-based approach and expert consensus from the spine oncology study group. Spine 35(22):E1221–E1229 10.1097/BRS.0b013e3181e16ae2 20562730

[21] Taori S, Adida S, Bayley JC, Zinn PO, Burton SA, Flickinger JC et al (2025) Long term outcomes following upfront stereotactic body radiotherapy alone for spinal metastases. J Neurooncol 175(1):243–254 10.1007/s11060-025-05126-6 40522562

[22] Adida S, Taori S, Sefcik RK, Burton SA, Flickinger JC, Gerszten PC (2025) Management for chordoma of the spine and sacrum with stereotactic radiosurgery. NeuroSurg Focus 58(5):E17 10.3171/2025.2.FOCUS24935 40311191

[23] Taori S, Adida S, Kann MR, Bhatia S, Sefcik RK, Burton SA et al (2025) Spine Stereotactic Radiosurgery Provides Long-Term Local Control and Overall Survival for Benign Intradural Tumors. Neurosurgery 96(5):1113–1123 10.1227/neu.0000000000003219 39440874

[24] Bhatia S, Taori S, Adida S, Kann MR, Rajan A, Burton SA et al (2026) The role of spinal stereotactic radiosurgery in the treatment of renal cell carcinoma spinal metastases. J Neurosurgery: Spine 44(6):952–962 10.3171/2025.11.SPINE25946 41871408

[25] National Cancer Institute (2017) Common terminology criteria for adverse events (CTCAE) version 5.0. U.S. Department of Health and Human Services, National Institutes of Health

[26] Dabbagh Ohadi MA, Delbari P, Ahmadvand MH, Zamani R, Karimi A, Ohadi N et al (2025) Single-fraction versus multifraction stereotactic radiosurgery for spinal metastases: Systematic review and meta-analysis. NeuroSurg Focus 58(5):E16 10.3171/2025.2.FOCUS24985 40311198

[27] Taori S, Adida S, Bhatia S, Kann MR, Rajan A, Choi S et al (2026) Stereotactic Body Radiotherapy for Spinal Metastases: Outcomes and Development of a Risk Assessment Score to Predict Local Tumor Progression. Neurosurgery. 10.1227/neu.0000000000003998 41860238

[28] Guninski RS, Cuccia F, Alongi F, Andratschke N, Belka C, Bellut D et al (2024) Efficacy and safety of SBRT for spine metastases: A systematic review and meta-analysis for preparation of an ESTRO practice guideline. Radiother Oncol 190:109969 10.1016/j.radonc.2023.109969 37922993

[29] Glicksman RM, Tjong MC, Neves-Junior WFP, Spratt DE, Chua KLM, Mansouri A et al (2020) Stereotactic ablative radiotherapy for the management of spinal metastases: A review. JAMA Oncol 6(4):567–577 10.1001/jamaoncol.2019.5351 31895403

[30] Zeng KL, Sahgal A, Husain ZA, Myrehaug S, Tseng CL, Detsky J et al (2021) Local control and patterns of failure for Radioresistant spinal metastases following stereotactic body radiotherapy compared to a Radiosensitive reference. J Neurooncol 152(1):173–182 10.1007/s11060-020-03691-6 33453002

[31] Taunk NK, Spratt DE, Bilsky M, Yamada Y (2015) Spine Radiosurgery in the Management of Renal Cell Carcinoma Metastases. J Natl Compr Canc Netw 13(6):801–809 10.6004/jnccn.2015.0093 26085394

[32] Bishop AJ, Tao R, Guadagnolo BA, Allen PK, Rebueno NC, Wang XA et al (2017) Spine stereotactic radiosurgery for metastatic sarcoma: Patterns of failure and radiation treatment volume considerations. J Neurosurgery: Spine 27(3):303–311 10.3171/2017.1.SPINE161045 28644075

[33] Gerszten PC, Burton SA, Quinn AE, Agarwala SS, Kirkwood JM (2005) Radiosurgery for the treatment of spinal melanoma metastases. Stereotact Funct Neurosurg 83(5–6):213–221 10.1159/000091952 16534253

[34] Adida S, Taori S, Tirmizi Z, Bayley JC, Zinn PO, Flickinger JC et al (2025) Stereotactic body radiation therapy for spinal metastases from gastrointestinal primary cancers. J Neurooncol 173(3):683–694 10.1007/s11060-025-05033-w 40227554

[35] Hashmi A, Guckenberger M, Kersh R, Gerszten PC, Mantel F, Grills IS et al (2016) Re-irradiation stereotactic body radiotherapy for spinal metastases: A multi-institutional outcome analysis. J Neurosurgery: Spine 25(5):646–653 10.3171/2016.4.SPINE151523 27341054

[36] Detsky JS, Nguyen TK, Lee Y, Atenafu E, Maralani P, Husain Z et al (2020) Mature Imaging-Based Outcomes Supporting Local Control for Complex Reirradiation Salvage Spine Stereotactic Body Radiotherapy. Neurosurgery 87(4):816–822 10.1093/neuros/nyaa109 32374852

[37] Taori S, Adida S, Choi S, McCaw TJ, Burton SA, Flickinger JC et al (2026) Repeat Stereotactic Body Radiation Therapy for Spinal Metastases: Long-Term Outcomes and Toxicity. Neurosurgery 99(1):76–86 10.1227/neu.0000000000003772 41055338

[38] Myrehaug S, Sahgal A, Hayashi M, Levivier M, Ma L, Martinez R et al (2017) Reirradiation spine stereotactic body radiation therapy for spinal metastases: Systematic review. J Neurosurgery: Spine 27(4):428–435 10.3171/2017.2.SPINE16976 28708043

[39] Jackson CB, Zhang L, Haseltine J, Mueller BA, Schmitt AM, Vaynrub M et al (2026) Reirradiation With Three-Fraction Stereotactic Body Radiation Therapy for Spinal Metastases. Practical Radiation Oncol 16(4):e293–e300 10.1016/j.prro.2026.01.015 PMC13527741 41786078

[40] Maranzano E, Bellavita R, Rossi R, De Angelis V, Frattegiani A, Bagnoli R et al (2005) Short-course versus split-course radiotherapy in metastatic spinal cord compression: Results of a phase III, randomized, multicenter trial. J Clin Oncol 23(15):3358–3365 10.1200/JCO.2005.08.193 15738534

[41] Klekamp J, Samii H (1998) Surgical results for spinal metastases. Acta Neurochir 140(9):957–967 10.1007/s007010050199 9842434

[42] Terzi S, Griffoni C, Rosa S, Cini C, Asunis E, Alcherigi C et al (2025) Health-related quality of life after surgery for spinal metastases. J Bone Oncol 52:100675 10.1016/j.jbo.2025.100675 PMC11986255 40226045

[43] Choi D, Fox Z, Albert T, Arts M, Balabaud L, Bunger C et al (2016) Rapid improvements in pain and quality of life are sustained after surgery for spinal metastases in a large prospective cohort. Br J Neurosurg 30(3):337–344 10.3109/02688697.2015.1133802 26901574

[44] Rothrock RJ, Barzilai O, Reiner AS, Lis E, Schmitt AM, Higginson DS et al (2021) Survival Trends After Surgery for Spinal Metastatic Tumors: 20-Year Cancer Center Experience. Neurosurgery 88(2):402–412 10.1093/neuros/nyaa380 PMC7803433 32970144

[45] Yamada Y, Katsoulakis E, Laufer I, Lovelock M, Barzilai O, McLaughlin LA et al (2017) The impact of histology and delivered dose on local control of spinal metastases treated with stereotactic radiosurgery. NeuroSurg Focus 42(1):E6 10.3171/2016.9.FOCUS16369 PMC5568686 28041329

[46] Owusuaa C, Dijkland SA, Nieboer D, van der Heide A, van der Rijt CCD (2022) Predictors of Mortality in Patients with Advanced Cancer-A Systematic Review and Meta-Analysis. Cancers 14(2):328 10.3390/cancers14020328 PMC8774229 35053493

[47] Salpeter SR, Malter DS, Luo EJ, Lin AY, Stuart B (2012) Systematic review of cancer presentations with a median survival of six months or less. J Palliat Med 15(2):175–185 10.1089/jpm.2011.0192 22023378

[48] Chao ST, Koyfman SA, Woody N, Angelov L, Soeder SL, Reddy CA et al (2012) Recursive partitioning analysis index is predictive for overall survival in patients undergoing spine stereotactic body radiation therapy for spinal metastases. Int J Radiat Oncol Biol Phys 82(5):1738–1743 10.1016/j.ijrobp.2011.02.019 21489717

[49] Sahgal A, Atenafu EG, Chao S, Al-Omair A, Boehling N, Balagamwala EH et al (2013) Vertebral compression fracture after spine stereotactic body radiotherapy: A multi-institutional analysis with a focus on radiation dose and the spinal instability neoplastic score. J Clin Oncol 31(27):3426–3431 10.1200/JCO.2013.50.1411 PMC6076012 23960179

[50] Kim YR, Lee CH, Yang SH, Hyun SJ, Kim CH, Park SB et al (2021) Accuracy and precision of the spinal instability neoplastic score (SINS) for predicting vertebral compression fractures after radiotherapy in spinal metastases: A meta-analysis. Sci Rep 11(1):5553 10.1038/s41598-021-84975-3 PMC7947012 33692442

[51] Taori S, Adida S, Bhatia S, Bayley JC, Zinn PO, Burton SA et al (2026) Risk Factors for Vertebral Compression Fracture Following Spine Stereotactic Body Radiation Therapy. Adv Radiation Oncol 11(6):102033 10.1016/j.adro.2026.102033 PMC13140028 42094940

[52] Patel PP, Esposito EP, Zhu J, Chen X, Khan M, Kleinberg L et al (2024) Antiresorptive Medications Prior to Stereotactic Body Radiotherapy for Spinal Metastasis are Associated with Reduced Incidence of Vertebral Body Compression Fracture. Global Spine J 14(6):1778–1785 10.1177/21925682231156394 PMC11268289 36749660

[53] Ozdemir Y, Torun N, Guler OC, Yildirim BA, Besen AA, Yetisken AG et al (2019) Local control and vertebral compression fractures following stereotactic body radiotherapy for spine metastases. J Bone Oncol 15:100218 10.1016/j.jbo.2019.100218 PMC6378905 30815342

[54] Chou KN, Park DJ, Sanker V, Ye X, Hori YS, Persad AR et al (2025) Vertebral fracture following primary stereotactic body radiation therapy for spinal bone metastases: A decade of experience. J Neurosurgery: Spine 43(3):294–304 10.3171/2025.3.SPINE231234 40577849

[55] Barzilai O, DiStefano N, Lis E, Yamada Y, Lovelock DM, Fontanella AN et al (2018) Safety and utility of kyphoplasty prior to spine stereotactic radiosurgery for metastatic tumors: A clinical and dosimetric analysis. J Neurosurgery: Spine 28(1):72–78 10.3171/2017.5.SPINE1746 PMC8274655 29087812

[56] Adida S, Kann MR, Taori S, Bhatia S, Sefcik RK, Burton SA et al (2025) Safety and Efficacy of Balloon-Assisted Kyphoplasty Followed by Stereotactic Body Radiation Therapy for Pathological Fractures. Neurosurgery 96(4):860–869 10.1227/neu.0000000000003182 39283076

[57] Adida S, Taori S, Rabon W, Bayley JC, Zinn PO, Agarwal N et al (2026) The 5-factor modified frailty index predicts local control and overall survival following spine stereotactic body radiation therapy. J Neurooncol 177(1). 10.1007/s11060-026-05488-5 41762350

